# Fabrication and Compression Properties of Two-Layered Porous Structure of Different Materials by Direct Printing of Resin Porous Structure on Aluminum Foam Using a 3D Printer

**DOI:** 10.3390/ma18020433

**Published:** 2025-01-17

**Authors:** Yoshihiko Hangai, Reiji Yamazaki, Takaaki Suzuki

**Affiliations:** Graduate School of Science and Technology, Gunma University, Kiryu 376-8515, Japan

**Keywords:** cellular materials, composite, 3D printing, foam, functionally graded

## Abstract

The porous structure, in which many pores are intentionally placed inside the material, has excellent impact energy absorption properties. Recent studies have attempted to fabricate multi-layered porous structures with different mechanical properties within a single porous structure sample, and the mechanical properties of these structures are being elucidated. However, these studies mainly attempted to vary the densities, pore structures, and alloy compositions within a single material, such as aluminum, for the entire sample. Since multi-materials are now being promoted to utilize the most suitable material type in the right place, porous structures made of different materials, such as a combination of aluminum and resin, are expected to be required in the future. In this study, we attempted to fabricate two-layered porous structure samples of different materials by printing a resin porous structure using a 3D printer on an aluminum foam fabricated by a precursor foaming process. Static compression tests were performed on the resulting two-layered porous structure samples to investigate their mechanical properties. The resin porous structure printed by the 3D printer and the aluminum foam were both designed to expose the porous structure on the surface of the specimen so that the deformation behavior can be easily observed. The density of the resin porous structure was varied by systematically varying the filling rate of the resin porous structure to be printed, and the effect on the compression properties was investigated. The fabricated two-layered porous structure was effectively bonded between the two layers by the anchor effect, which is a mechanical bonding caused by the resin penetrating into the pores. The layers exhibited robust bonding with no evidence of separation. It was possible to fabricate a two-layered porous structure that exhibited both properties of aluminum foam and those of resin porous structure. It was found that the plateau stress in the resin porous structure layer can be controlled between about 0.5 MPa and 40 MPa, and the deformation behavior and energy absorption properties of the two-layered porous structure can be controlled by varying the resin filling rate of the resin porous structure layer. That is, it was indicated that multi-layered porous structures with various densities and consisting of various types of materials allow for the optimal design of porous structures used in structural materials.

## 1. Introduction

The porous structure, in which many pores are intentionally placed inside the material, has excellent impact energy absorption properties. Therefore, it is used as a shock-absorbing component to improve the safety of automobiles and as a landing device for space vehicles [1,2,3,4,5,6,7]. Recent studies have attempted to fabricate multi-layered porous structures with different mechanical properties within a single porous structure sample, and the mechanical properties of these structures are being elucidated [8,9,10]. In multi-layered porous structures using aluminum as the base material, the direct foaming of molten aluminum [11,12,13] has been used to fabricate graded pore structures of aluminum foams by varying the cooling rate [14,15,16,17]. In the syntactic foam [18,19], graded pore structures of aluminum syntactic foams were fabricated by varying the type and amount of particles [20,21,22,23]. In the powder space holder technique, in which the powder and spacer are sintered, and then the foams are fabricated by removing the spacer from the sintered compact [24,25,26,27], aluminum foams with gradient pore structures and alloy compositions have been fabricated [28,29,30,31]. By applying the precursor foaming method [32,33], graded aluminum foams with varying densities and alloy types have been fabricated [34,35,36]. Furthermore, a two-layer porous structure in which both open-cell and closed-cell structures coexist has been developed by combining both the powder space holder technique and the precursor foaming method [37]. Many resin matrix materials with graded porous structures and graded honeycombs have been fabricated using a 3D printer with varying densities by varying filling rates of resin [38,39,40,41,42,43,44]. The use of a 3D printer makes it possible to achieve the desired pore structures with high precision [45], making it easy to achieve the desired properties of each layer. For example, Bates et al. [38] fabricated a three-layered porous resin using a 3D printer and found that three plateau regions appeared, each with constant stress, during static compression tests.

Although many multi-layered porous structures have been developed, as mentioned above, the main attempt is to vary the densities, pore structures, and alloy compositions within a single material, such as aluminum, for the entire sample. However, since multi-materials are now being promoted to utilize the most suitable material type in the right place [46,47,48], porous structures made of different materials, such as a combination of aluminum and resin, are expected to be required in the future, but few studies have been conducted. Recently, attempts have been made to bond dense resin to metal foam, including thermocompression bonding [49,50], incremental forming [51,52], and friction welding [53,54]. In our previous study [55], we attempted to print resin directly on aluminum foam using a 3D printer. It was found that the resin can penetrate into the pores of the aluminum foam and bond it due to the anchor effect. However, the mechanical properties of the fabricated samples had not been investigated.

In this study, therefore, we attempted to fabricate two-layered porous structure samples of different materials by printing a resin porous structure using a 3D printer on an aluminum foam fabricated by a precursor foaming process. Static compression tests were performed on the resulting two-layered porous structure samples to investigate their mechanical properties. The resin porous structure printed by the 3D printer and the aluminum foam were both designed to expose the porous structure on the surface of the specimen so that the deformation behavior can be easily observed. The density of the resin porous structure was varied by systematically varying the filling rate of the resin porous structure to be printed, and the effect on the compression properties was investigated.

## 2. Materials and Methods

### 2.1. Fabrication of Aluminum Foam

**Figure 1:**
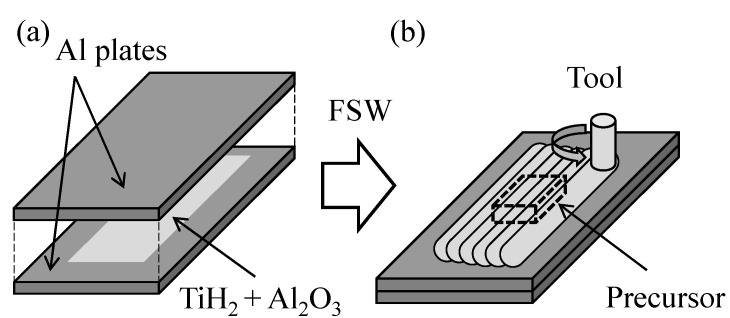
Schematic illustration of precursor fabrication. (**a**) A laminate plate was made by placing a foaming agent powder and a pore structures stabilizer powder between two aluminum alloy plates. (**b**) The FSW tool was traversed over the laminate plate.

The aluminum foams used in this study were fabricated by the precursor foaming method using friction stir welding (FSW) [56,57,58,59,60]. Figure 1 shows a schematic illustration of precursor fabrication. As shown in Figure 1a, a laminate plate was made by placing a foaming agent powder and a pore structure stabilizer powder between two aluminum alloy plates. Al-Si-Cu ADC12 aluminum alloy die-casting plates (210 mm × 80 mm × 3 mm thickness) were used as aluminum alloy plates, titanium hydride powder (TiH_2_, particle diameter less than 45 μm) was used as a foaming agent, and alumina powder (Al_2_O_3_, particle diameter about 1 μm) was used as a pore structures stabilizer. The FSW tool was traversed over the laminate plate as shown in Figure 1b, and the powders were mixed into the ADC12 plates during the welding of the two plates. The rotating speed of the FSW tool was 1200 rpm, the tool traversing rate was 120 mm/min, and the tilt angle was 3°. The FSW tool was made of tool steel with a shoulder diameter of 17 mm, and the probe was M6 threaded and 4.8 mm long. Since a large-area precursor cannot be fabricated in a single FSW tool traversing, six FSW tool traverses were performed, shifting 5 mm per tool traversing perpendicularly to the traversing direction. Since one tool traversing was insufficient for mixing powders into ADC12 plates, the same area was repeatedly traversed several times; that is, the FSW tool was traversed for a total of six rows × four times. These FSW conditions were performed in a similar way, referring to the literature [54]. A 30 mm × 18 mm × 6 mm thickness precursor was machined from the tool-traversed area.

**Figure 2:**
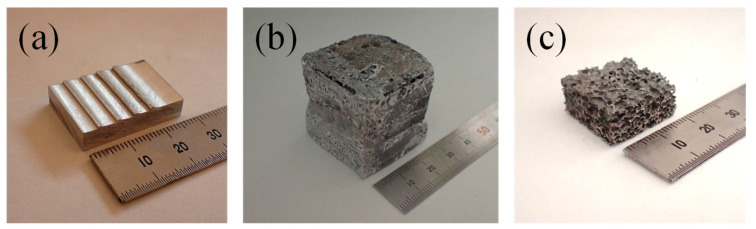
Obtained sample. (**a**) Precursor. (**b**) Foamed sample. (**c**) Aluminum foam specimen.

Figure 2a shows the precursor actually obtained. Two precursors were then placed in a32 mm × 32 mm × 30 mm height steel molds and foamed by optical heating [61] with three halogen lamps (line heaters with a maximum output of 2 kW) each from both the top and bottom sides. Each halogen lamp operated at a voltage of 160 V and a current of 8 A. Figure 2b shows the sample actually foamed. A 20 mm × 20 mm × 10 mm height aluminum foam specimen, as shown in Figure 2c, was cut by electrical discharge machining from the foamed sample.

### 2.2. Resin Printing on Aluminum Foam

**Figure 3:**
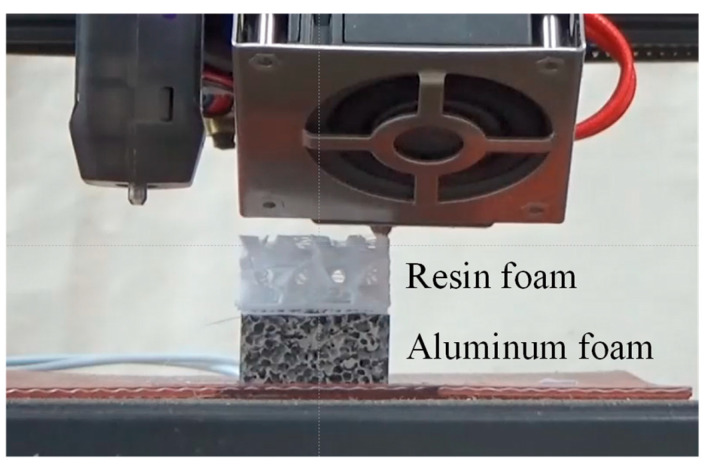
Resin porous structure was printed on the aluminum foam sample using a 3D printer.

The resin porous structure was printed on the aluminum foam sample prepared in Section 2.1 using a 3D printer. Figure 3 shows the actual printing process. The lower part is an aluminum foam layer, and the upper white part is a resin porous structure layer. The 3D printer was an Ender3 S1 Pro (manufactured by Creality, Shenzhen, China), and polylactic acid (PLA) resin was used as filament. To print the resin on the aluminum foam in the softest possible state and to facilitate penetration of the resin into the pores of the aluminum foam, the temperature of the 3D printer plate was set to 100 °C, the highest temperature that can be set. A heater that can raise the temperature to a maximum of 200 °C was placed on the 3D printer plate, and the aluminum foam was fixed on it with double-sided tape and left for 30 min before printing began. The temperature of the resin during printing was set at 230 °C, which is the highest temperature that can be set by the 3D printer used in this study. Printing was performed at a thickness of 0.2 mm per layer and a printing speed of 50 mm/s. The resin filling rate *φ* was varied from 10% to 80% in 10% increments and printed on the aluminum foam, respectively. Note that, on the bonding surface with aluminum foam (the bottom surface of the resin porous structure layer), the first four layers (0.8 mm thickness) were made dense with a filling rate of 100% to allow more resin to penetrate into the pores, and a porous structure with the specified filling rate was applied from the fifth layer. Furthermore, the 3D printer used in this study cannot apply a pressing force when printing. Therefore, the nozzle was placed as close as possible to the aluminum foam so that the resin could easily penetrate into the pores, and a gap of about 0.1 mm thickness was set. These conditions were performed in a similar way, referring to the literature [55] in which resin was printed directly on aluminum foam using a 3D printer.

**Figure 4:**
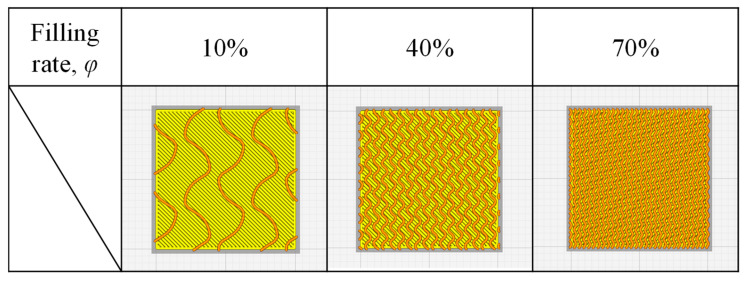
Cross-section of the printing data input to the 3D printer for the resin filling rate *φ* = 10%, 40%, and 70% to print a resin porous structure.

Figure 4 shows a cross-section of the printing data input to the 3D printer for the resin filling rate *φ* = 10%, 40%, and 70% to print a resin porous structure. It can be seen that the density is low when *φ* is low and that the density increases as *φ* increases because there are more resin parts.

A porous structure of 20 mm × 20 mm × 10 mm height with the same dimensions as the aluminum foam sample was thus printed on top of the aluminum foam sample. Two compression test specimens of 20 mm × 20 mm × 20 mm, including the aluminum foam layer, were prepared for each resin filling rate *φ*. For comparison, two 20 mm × 20 mm × 10 mm height compression specimens of the uniform resin porous structure were also prepared for each *φ*.

### 2.3. Compression Test

Compression tests were conducted using a universal testing machine (Instron 5582, Norwood, MA, USA). Deformation behavior during the test was captured by a video camera. The compression test rate was 1.2 mm/min. The compression stress *σ* and the compression strain *ε* were obtained from the load and displacement output from the testing machine. *σ* was obtained by dividing the load by the initial cross-sectional area (20 mm × 20 mm), and *ε* was obtained by dividing the displacement by the initial height (20 mm for the two-layered porous structure and 10 mm for the uniform resin porous structure).

## 3. Results and Discussion

### 3.1. Obtained Compression Test Specimen

**Figure 5:**
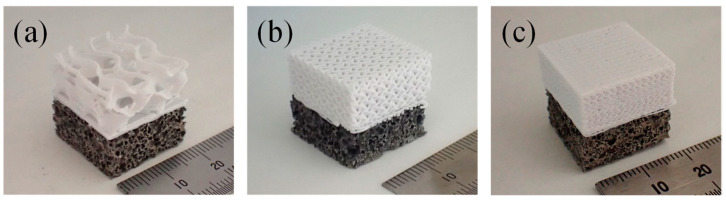
Obtained two-layered porous structure compression specimens with filling rates of (**a**) *φ* = 10%, (**b**) *φ* = 40%, and (**c**) *φ* = 70%, for the resin porous structure layer.

Figure 5 shows typical examples of compression specimens with filling rates of *φ* = 10%, 40%, and 70% for the resin porous structure layer, respectively. The lower part is an aluminum foam layer, and the upper white part is a resin porous structure layer. It was shown that a resin porous structure can be printed on aluminum foam using a 3D printer, resulting in compression specimens with two-layered porous structures. No separation between the two layers was observed. Compression specimens with a resin porous structure layer of other *φ* can also be printed while bonding the resin porous structure layer on top of the aluminum foam layer in a similar process, and compression specimens with two-layered porous structures can be fabricated regardless of the *φ*.

### 3.2. Compression Test Results of Uniform Resin Porous Structure

**Figure 6:**
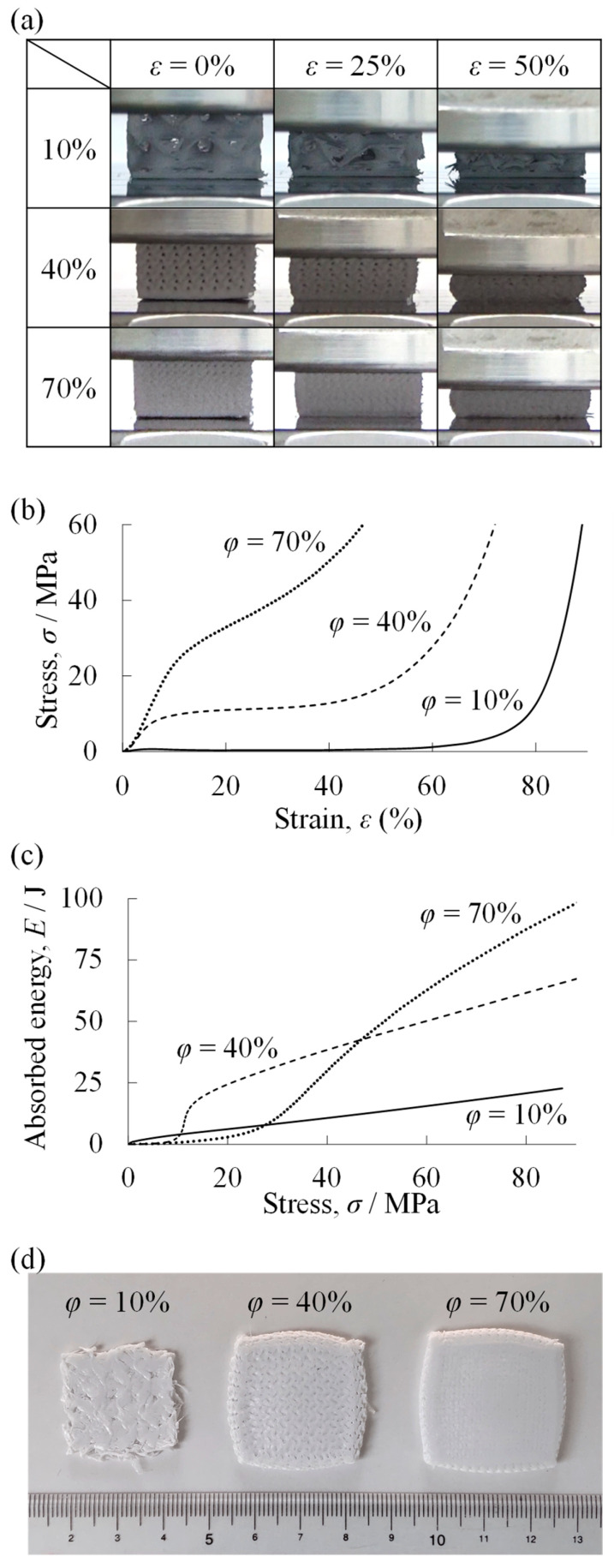
Results of compression test of uniform resin porous structures. (**a**) Compression deformation behaviors. (**b**) Stress *σ*–strain *ε* curves. (**c**) Relationship between stress and absorbed energy. (**d**) Specimens after compression tests.

Figure 6a shows the compression deformation behaviors of resin porous structure specimens with filling rates of *φ* = 10%, 40%, and 70%. The uniform PLA resin porous structure deformed ductilely at all *φ*, without showing a ragged collapsing deformation behavior.

Figure 6b shows the *σ*–*ε* curves for these compression deformation behaviors. Generally, the *σ*–*ε* curve of a foam has three regions: first, an elastic region where stress increases linearly at low *ε* in the early stages of the compression test, then a plateau region where stress is almost constant and strain increases due to laminar deformation, and finally a densification region where the entire specimen becomes denser and stress rises sharply at high *ε* in the final stages of the compression test [62,63,64]. In the uniform resin porous structure specimens in this study, three regions appeared as in ordinary foams: an elastic region, a plateau region, and a densification region. The plateau stress [64,65], which is the average of the stresses at *ε* = 20–30%, increased as the *φ* of the resin porous structure specimen increased, indicating higher strength. The rate of increase in stress was also higher in the plateau region for the *φ* = 70% resin porous structure specimen.

Figure 6c shows the relationship between stress and absorbed energy obtained from the *σ*–*ε* curves of uniform resin porous structures. This absorbed energy is the accumulated energy absorbed until a certain stress is reached. The resin porous structure with *φ* = 10% had the highest absorbed energy up to around *σ* = 10 MPa. On the other hand, around *σ* = 10–45 MPa, the resin porous structure with *φ* = 40% had the advantage in absorbed energy, and above *σ* = 45 MPa, the resin porous structure with *φ* = 70% had the highest absorbed energy. That is, depending on the *σ* that the structure is subjected to, there is an optimum *φ* resin porous structure that can absorb a large amount of energy.

Figure 6d shows the resin porous structure specimens after the compression test with filling rates of *φ* = 10%, 40%, and 70%. As shown in the deformation behavior section of Figure 6a, there were no debris, indicating that the resin porous structures in this study deformed ductilely.

### 3.3. Compression Test Results of Two-Layered Porous Structure

**Figure 7:**
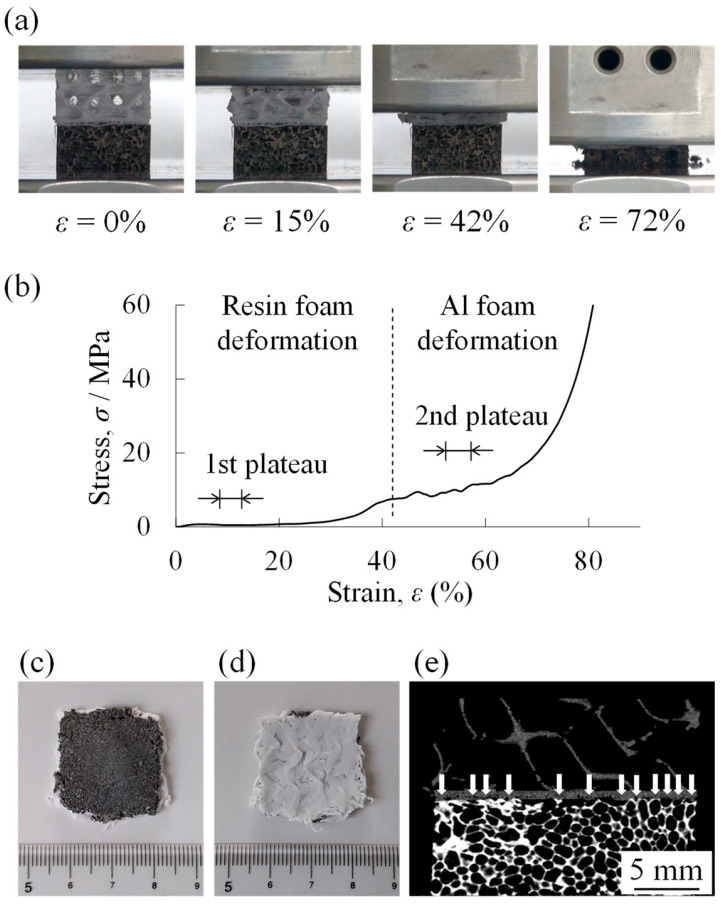
Results of compression test of a two-layered porous structure compression specimen with a filling rate of *φ* = 10% in the resin porous structure layer. (**a**) Compression deformation behavior. (**b**) Stress *σ*–strain *ε* curve. (**c**) Specimen of aluminum foam layer side after compression test. (**d**) Specimen of resin porous structure layer side after compression test. (**e**) X-ray CT image of specimen before compression test. The white arrows indicate where PLA penetration can be observed in the pores.

Figure 7a shows the compression deformation behavior of a two-layered porous structure compression specimen with a filling rate of *φ* = 10% in the resin porous structure layer. The lower part was an aluminum foam layer, and the upper part was a resin porous structure layer. When the compression test started, deformation began first in the upper resin porous structure layer. The resin porous structure layer deformed ductilely. When the deformation of the resin porous structure layer was almost completed, the deformation of the lower aluminum foam layer began at around *ε* = 40%. This indicates that the aluminum foam layer was higher in strength than the resin porous structure layer with *φ* = 10%. The aluminum foam layer was deformed in a brittle manner. This is due to the use of an Al-Si-Cu aluminum alloy with a brittle eutectic structure [66,67] in the matrix, which is consistent with the deformation behavior of Al-Si-Cu aluminum foam in the literature [35,68]. The *σ*–*ε* curve obtained from this compression test is shown in Figure 7b. First, an elastic region (at *ε* lower than approximately 5%) and a plateau region appeared, then the stress began to increase at around *ε* = 35% and the plateau region appeared again, and finally a densification region appeared at around *ε* = 70%. That is, in the case of a two-layered porous structure, the two plateau regions appeared. Referring to the compression deformation behavior in Figure 7a, it can be seen that the 1st plateau region corresponded to the deformation of the resin porous structure layer and the 2nd plateau region corresponded to the deformation of the aluminum foam layer. In particular, the stress in the 1st plateau region was almost the same as that of the plateau region of the uniform resin porous structure with *φ* = 10% shown in Figure 6b. The stress oscillation during the deformation of the aluminum foam layer is due to the brittle deformation of the aluminum foam layer, which was fabricated from Al-Si-Cu aluminum alloy.

Figure 7c and Figure 7d show the specimen after the compression test of the aluminum foam layer side and the resin porous structure layer side, respectively. No delamination was observed between the aluminum foam layer and the resin porous structure layer during the compression test. This phenomenon was similar regardless of the *φ* of the resin porous structure layer. Figure 7e shows an X-ray CT image of the specimen before the compression test. The upper layer was the resin porous structure layer, and the lower layer was the aluminum foam layer. As indicated by the white arrows, resin has penetrated into the pores of the aluminum foam in the vicinity of the bonding interface. This suggests that the anchor effect caused deformation without delamination between the resin porous structure layer and the aluminum foam layer.

**Figure 8:**
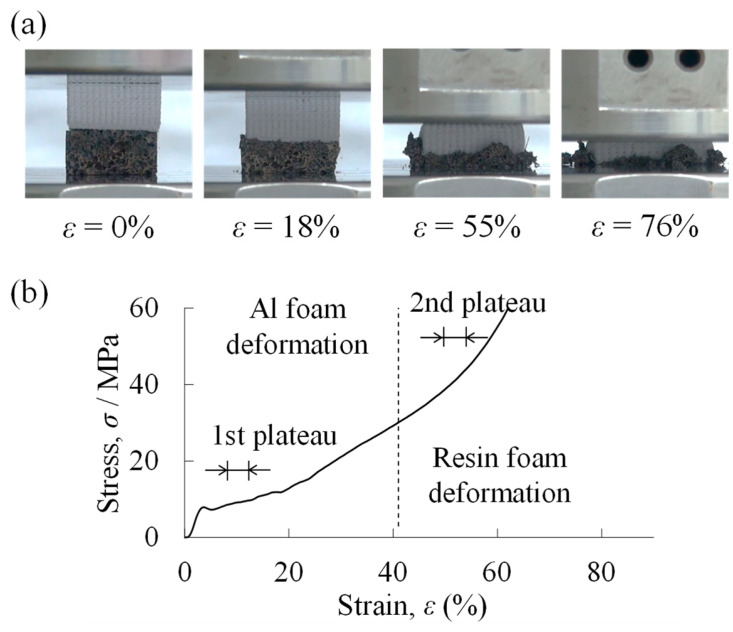
Results of compression test of a two-layered porous structure compression specimen with a filling rate of *φ* = 70% in the resin porous structure layer. (**a**) Compression deformation behavior. (**b**) Stress *σ*–strain *ε* curve.

Figure 8a shows the compression deformation behavior of a two-layered porous structure compression specimen with a filling rate of *φ* = 70% in the resin porous structure layer. The lower part was an aluminum foam layer, and the upper part was a resin porous structure layer. When the compression test started, deformation began first in the aluminum foam layer at the bottom, and when the deformation was almost completed, the resin porous structure layer at the top began to deform around *ε* = 40%. This indicates that the *φ* = 70% resin porous structure layer was higher in strength than the aluminum foam layer. The *σ*–*ε* curve obtained from this compression test is shown in Figure 8b. First, an elastic region (at *ε* lower than approximately 5%) and a plateau region appeared, followed by a gradual increase in stress, and finally a densification region. Referring to the compression deformation behavior in Figure 8a, it can be seen that the initial plateau region corresponded to the deformation of the aluminum foam layer, and the region of gradually increasing stress corresponded to the plateau region of the resin porous structure layer. The resin porous structure layer with *φ* = 70% was close to the dense material, and the rate of increase in stress was higher in the plateau region, which was almost similar to the plateau region of the uniform resin porous structure with *φ* = 70% shown in Figure 6b.

**Figure 9:**
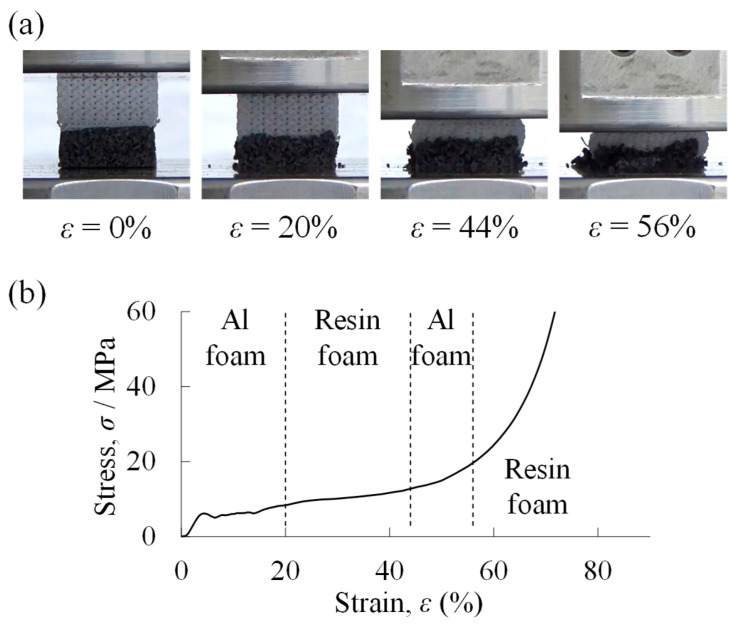
Results of compression test of a two-layered porous structure compression specimen with a filling rate of *φ* = 40% in the resin porous structure layer. (**a**) Compression deformation behavior. (**b**) Stress *σ*–strain *ε* curve.

Figure 9 shows the compression deformation behavior and the *σ*–*ε* curve of a two-layered porous structure compression test in which the filling rate of the resin porous structure layer was *φ* = 40%. First, deformation started from the aluminum foam layer, but only a partial deformation occurred, then the resin porous structure layer started to deform and became dominant. Then, when a part of the resin porous structure layer finished its deformation, the deformation of the aluminum foam layer became dominant again, and finally the deformation of the resin porous structure layer became dominant. The *σ*–*ε* curve shows only one plateau region, as in a conventional foam, instead of two distinct plateau regions as shown in Figure 7 and Figure 8. This can be attributed to the fact that the strength of both the aluminum foam layer and the resin porous structure layer was nearly constant.

Consequently, the order of deformation can be arbitrarily controlled, and the plateau stress value can be modified by varying the filling rate of the resin porous structure layer. In contrast, when the strength difference between the upper and lower layers is less, deformation can occur alternately, resulting in the appearance of almost a single plateau stress, as in a uniform porous structure.

### 3.4. Plateau Stress Estimation Method for Two-Layered Porous Structure

In this section, the plateau stress was estimated from the obtained *σ*–*ε* curves. In the case of a compression test of a uniform porous structure, the plateau stress is defined by the average stress for *ε* = 20–30%, according to the literature [64,65]. In the case of a two-layered porous structure, it was difficult to estimate the plateau stress using the same definition as for a uniform porous structure. Therefore, we assumed that the deformation between the start of deformation of the first layer and the start of deformation of the second layer was that of only the first layer. And we assumed that the strain during this deformation of the first layer was 100%, and the average stress corresponding to 20–30% of the strain was defined as the plateau stress in the first layer. The deformation of the second layer was assumed to be from the start of the deformation of the second layer to the end of the test. The strain during the deformation of the second layer was assumed to be 100%, and the average stress corresponding to 20–30% of the deformation was defined as the plateau stress of the second layer [34,35]. For example, in Figure 7, the deformation of the resin porous structure layer was assumed to be from *ε* = 0% to *ε* = 42%. Assuming the range *ε* = 0–42% to be 100%, the plateau stress is defined as the average of the stresses in the part of the range corresponding to 20–30%. Similarly, the deformation of the aluminum foam layer was assumed to be after *ε* = 42%, and the plateau stress of the aluminum foam layer was defined as the average value of the area corresponding to 20–30% strain. When the plateau stress was estimated based on the above assumptions, the plateau stress of the resin porous structure layer, which was the 1st plateau region, was 0.48 MPa, and the plateau stress of the aluminum foam layer, which was the 2nd plateau region, was 10.0 MPa. Similarly, in Figure 8, the plateau stress corresponding to the deformation of the aluminum foam layer, the 1st plateau region, was 9.16 MPa, and the plateau stress corresponding to the deformation of the resin porous structure layer, the 2nd plateau region, was 41.1 MPa. In the case of the alternating deformation of the porous resin and aluminum foam layers as shown in Figure 9, no clear two plateau regions appeared. Therefore, the *ε* = 20–30% part of the overall *σ*–*ε* curve, as in a conventional uniform foam, was defined as the common plateau stress for both layers, which in this case was 9.49 MPa.

**Figure 10:**
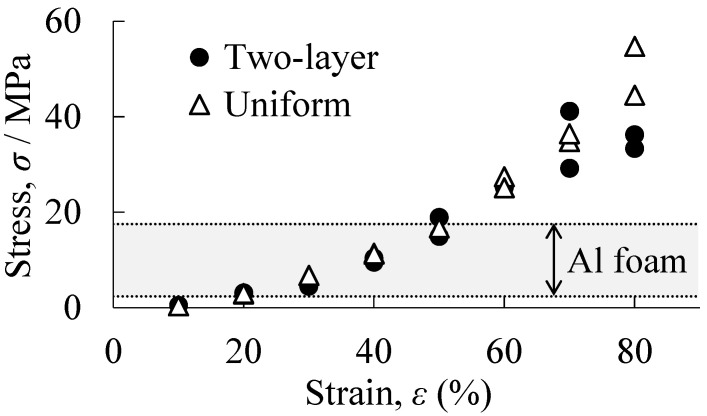
Plateau stresses of the resin porous structure in the two-layered porous structures and the uniform resin porous structures for each filling rate *φ*.

Figure 10 shows the plateau stresses of the resin porous structure in the two-layered porous structures and the uniform resin porous structures for each filling rate *φ*. The results were obtained from two trials for each. The range of plateau stresses in the aluminum foam layers of the two-layered porous structure obtained in this study is also shown in the banded range. It can be seen that the plateau stress exhibited by the resin porous structure layer of the two-layered porous structures and the plateau stress of the uniform resin porous structures were almost the same value. That is, in two-layered porous structures, by varying the filling rate of the resin porous structure layer, it is possible to exhibit the same properties as a uniform resin porous structure with each filling rate. Generally, aluminum foam varies in plateau stress due to the foaming process, and this was also the case in this study. It can be seen that when the *φ* of the resin porous structure layer was low, the resin porous structure layer had lower strength than the aluminum foam layer, and when the *φ* of the resin porous structure layer was high, the resin porous structure layer had higher strength than the aluminum foam layer. It can also be seen that there were *φ* in which the resin porous structure layer and the aluminum foam layer exhibited the same strength. Therefore, it was shown that by adjusting *φ*, the required strength can be achieved in the resin porous structure layer and the deformation can be controlled.

**Figure 11:**
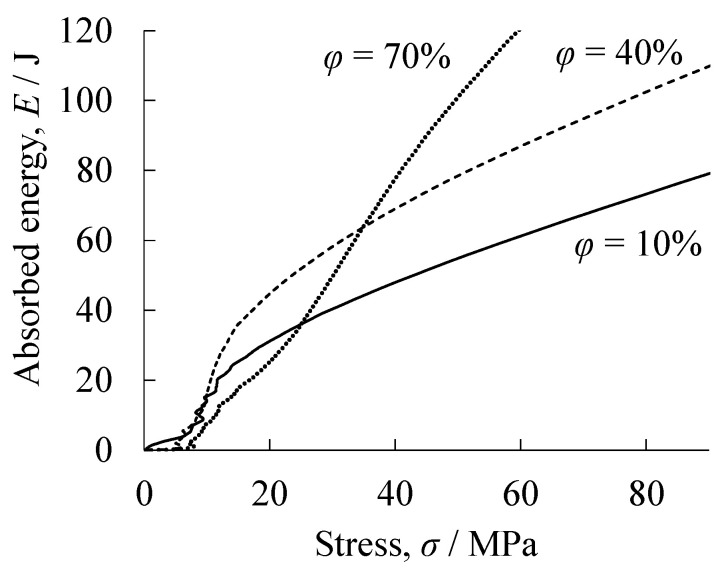
Relationship between stress and absorbed energy estimated from the *σ*–*ε* curves of the obtained two-layered porous structures.

Figure 11 shows the relationship between stress and absorbed energy estimated from the *σ*–*ε* curves of the obtained two-layered porous structures. Referring to the *σ*–*ε* curve in Figure 7b, for the two-layered porous structure with a *φ* = 10% resin porous structure layer, the *φ* = 10% resin porous structure layer absorbed energy up to around *σ* = 5 MPa. After exceeding *σ* = 5 MPa, where the energy absorption increased rapidly, the aluminum foam layer absorbed most of the energy. That is, for a two-layered porous structure with a *φ* = 10% resin porous structure layer, the resin porous structure layer can absorb energy at *σ* < 5 MPa, and the aluminum foam layer can absorb energy at higher stress. However, at high *σ*, the resin porous structure layer was almost completely compressed, thus it did not play an energy-absorbing role. In contrast, referring to the *σ*–*ε* curve in Figure 8b, for the two-layered porous structure with a *φ* = 70% resin porous structure layer, almost no energy can be absorbed up to around *σ* = 5 MPa. It is considered that the aluminum foam layer absorbed energy mainly after *σ* = 5 MPa, and that the resin porous structure layer with *φ* = 70% absorbed energy mainly after *σ* = 40 MPa. That is, the two-layered porous structure with a *φ* = 70% resin porous structure layer can absorb little energy at *σ* < 5 MPa, but the aluminum foam layer can absorb energy mainly at *σ* = 5–40 MPa, and at higher stresses, the resin porous layer can absorb energy. The two-layered porous structure with *φ* = 40% resin porous structure layer exhibited energy absorption properties between the above two two-layered porous structures. From these results, a two-layered porous structure combining a low *φ* resin porous structure layer and an aluminum foam layer is suitable for energy absorption at low stresses up to around *σ* = 5 MPa and for continued energy absorption at higher stresses. In contrast, if energy absorption is not required at low stresses, but rather energy absorption begins gradually around *σ* = 5 MPa and more energy is to be absorbed at higher stresses, a two-layered porous structure combining a high *φ* resin porous structure layer and an aluminum foam layer is found to be suitable. It was found that a multi-layered porous structure with various properties can absorb energy in various stress zones.

From these results, it is indicated that by combining porous structures with different compression properties, multiple compression properties can be achieved within a single porous structure. And it is suggested that the compression properties can be controlled by considering factors such as the density of the porous structure and the type of material. In this study, aluminum foam fabricated by the precursor method was used, but it is considered that aluminum foam fabricated by other methods can also exhibit the desired mechanical properties in combination with the resin porous structure. In particular, since porous structures of aluminum alloy can be fabricated by metal 3D printers in recent years [69,70,71,72], multi-layered porous structures consisting of aluminum porous structures and resin porous structures fabricated by 3D printers are expected to be possible in the future. The use of metal 3D printers allows precise control of the properties of the aluminum foam layers, and multi-layered porous structures with various densities and types of materials are expected to enable the optimal design of porous structures used in structural materials, such as shock absorbers for automobiles. If the resin part is deformed by a minor collision, it can be easily repaired by a 3D printer, thus extending the service life of the part.

## 4. Conclusions

In this study, we attempted to fabricate a two-layered porous structure of different materials by printing a resin porous structure by a 3D printer on an aluminum foam fabricated by the precursor foaming method. The following results were obtained from the experiments.

(1) In the uniform specimen of the resin porous structure fabricated by the 3D printer, three regions appeared: an elastic region, a plateau region, and a densification region, just as in conventional foams. The plateau stress increased as the resin filling rate *φ* of the uniform specimen of the resin porous structure increased, indicating that the specimen became higher in strength. The resin porous structure with small *φ* absorbed the maximum energy at low stress, while the resin porous structure with large *φ* absorbed the maximum energy at high stress. That is, the compression properties can be controlled by varying the resin filling rate of the uniform resin porous structure.
(2) The fabricated two-layered porous structure was effectively bonded between the two layers by the anchor effect, and no separation occurred. That is, it was found that a two-layered porous structure, in which the aluminum foam and the resin porous structure were combined, can be fabricated by directly printing the resin porous structure on the aluminum foam fabricated by the precursor method using a 3D printer.
(3) By fabricating a two-layered structure consisting of aluminum foam and a resin porous structure, it was possible to fabricate a porous structure that exhibited both properties of aluminum foam and those of a resin porous structure. It was found that the deformation behavior and energy absorption properties of the two-layered porous structure can be controlled by varying the resin filling rate of the resin porous structure layer. That is, it was indicated that multi-layered porous structures with various densities and consisting of various types of materials allow for the optimal design of porous structures used in structural materials.

## Data Availability

The original contributions presented in this study are included in the article. Further inquiries can be directed to the corresponding author.
